# Relationship between Serum Uric Acid Levels and Nonalcoholic Fatty Liver Disease in Non-Obese Patients

**DOI:** 10.3390/medicina55090600

**Published:** 2019-09-17

**Authors:** Alihan Oral, Tolga Sahin, Fatih Turker, Erdem Kocak

**Affiliations:** 1 Department of Internal Medicine, Faculty of Medicine, Demiroglu Bilim University, 34360 Istanbul, Turkey; fatihturker1985@hotmail.com; 2 Department of Gastroenterology, Faculty of Medicine, Demiroglu Bilim University, 34360 Istanbul, Turkey; kocak67@hotmail.com (E.K.); drtolgasahin@gmail.com (T.S.)

**Keywords:** liver, steatosis, NAFLD, uric acid

## Abstract

*Background and objectives:* Nonalcoholic fatty liver disease (NAFLD) is associated with multiple factors such as hypertension, diabetes, dyslipidemia, obesity, and hyperuricemia. We aim to investigate the relationship between uric acid and NAFLD in a non-obese and young population. *Materials and Methods:* This study was performed in January 2010–2019 with a group of 367 (225 patients in the NAFLD group and 142 in the control group) patients with liver biopsy-proven NAFLD or no NAFLD. Patients with NAFLD were classified according to the percentage of steatosis as follows, group I had 1–20% and group II >20%. Demographic, clinical, and laboratory (biochemical parameters) features were collected retrospectively. *Results:* The mean body mass index (BMI) and age of the patients were 26.41 ± 3.42 and 32.27 ± 8.85, respectively. The BMI, homeostatic model of assessment (HOMA-IR), and uric acid (UA) values of the NAFLD group were found to be significantly higher than those of the controls. A positive correlation was found between the NAFLD stage and UA. The following factors were independently associated with NAFLD: BMI, HOMA-IR, and UA. In addition, the cut-off value of UA was 4.75 mg/dl with a sensitivity of 45.8% and a specificity of 80.3%. *Conclusions:* UA is a simple, non-invasive, cheap, and useful marker that may be used to predict steatosis in patients with NAFLD.

## 1. Introduction

Nonalcoholic fatty liver disease (NAFLD) is defined as a condition where more than 5% of the body’s hepatic cells contain fatty deposits without a history of alcohol consumption. This deposition of fat can cause a variety of diseases ranging from simple steatosis to non-alcoholic steatohepatitis (NASH), cirrhosis, liver failure, and hepatocellular carcinoma [1]. NAFLD is diagnosed when daily alcohol consumption is ≤20 g/day in women and ≤30 g/day in men and other causes of the disease have been excluded (autoimmune, viral, steatogenic drugs, etc.) [2,3]. NAFLD is a public health problem worldwide. Recent studies show that NAFLD can be seen in both weak and obese populations [4]. According to epidemiological studies, 30% of the general population has steatosis, 5% of whom develop NASH [1]. NAFLD is considered to be associated with the expression of multi-metabolic disorders [5]. NAFLD is associated with multiple factors such as hypertension, insulin resistance, diabetes, dyslipidemia, obesity, and hyperuricemia [2,6,7,8].

Uric acid (UA) is a natural end product of purine metabolism. While it is naturally found in blood in certain amounts, its deviation can indicate a metabolic disorder. Lately, links have been suggested between the UA level and diabetes mellitus, obesity, cardiovascular disease, and renal disease [8,9,10,11,12,13,14]. Additionally, several studies have demonstrated a correlation between serum UA levels and NAFLD and NASH. This condition is thought to mostly occur in patients with obesity, insulin resistance, and metabolic syndrome. [8,9,10,11,12]. Unfortunately, these studies lack the ability to elaborate on the causal aspect of this correlation. Besides UA levels being elevated due to factors that cause NAFLD, hyperuricemia itself is a proven cause of NAFLD, independent of obesity or insulin resistance [15]. In some studies, it was found that uric acid has effects such as causing inflammation and nitric oxide inhibition when it enters endothelial and fat cells [16].

In this study, the association between serum UA and the severity of liver damage was evaluated in a large cohort of biopsy-proven, non-obese, and young Turkish individuals with NAFLD.

## 2. Materials and Methods

This study was performed in a group of 367 patients with liver-biopsy-proven NAFLD or no NAFLD between January 2010 and January 2019 at the Istanbul Demiroglu Bilim University Gastroenterology Department. The study population included 225 patients with biopsy-proven NAFLD and 142 control subjects without NAFLD. NAFLD was diagnosed by liver biopsy. Liver histology was evaluated by an experienced liver pathologist, and specimens were classified according to the NASH Clinical Research Network guidelines [17]. Patients with NAFLD were classified according to the percentage of steatosis as follows, group I had 1–20% and group II >20%. The study was approved by the local ethics committee (approval number 2019-16-03; approved on 08.06.2019).

The inclusion criteria were for male or female individuals aged between 18 and 65 years with no history of excessive alcohol drinking (considered as an average daily consumption of alcohol >30 g/day in men and >20 g/day in women), negative tests for the presence of the hepatitis B surface antigen and the antibody to hepatitis C virus, absence of a history of cirrhosis and other causes of liver disease (hemochromatosis, autoimmune hepatitis, Wilson’s disease), and no treatment with drugs that are known to cause liver steatosis (e.g., corticosteroids, estrogens, methotrexate, tetracycline, calcium channel blockers, or amiodarone). The exclusion criteria were the presence of any other chronic liver disease, HIV infection, diabetes mellitus, heart failure, valvular disease, asthma, chronic obstructive pulmonary disease, peripheral and cerebral vascular disease, hematological disorders, acute or chronic infections, cancer history involving liver transplantation, previous exposure to drugs associated with fatty liver, or refusal to participate in the study. Demographic and anthropometric data were obtained (age, gender, weight, height, and body mass index (BMI)) and serum biochemistry was assessed including total cholesterol, triglyceride, alanine aminotransferase (ALT), aspartate aminotransferase (AST), gamma glutamyl transferase (GGT), alkaline phosphatase (ALP), total bilirubin, albumin, blood urea nitrogen (BUN), creatinine, and UA concentrations. The evaluation of insulin resistance relied on the homeostatic model of assessment (HOMA-IR) calculation (Fasting insulin (mU/mL) × glucose (mmol/L) / 22.5). A score of HOMA-IR ≥2.5 was used to define insulin resistance [18]. All biochemical parameters were received after 8 hours of angle and were measured on a multichannel autoanalyzer (Hitachi Inc, Tokyo, Japan).

Data are expressed as the mean ± standard deviation. A statistical analysis was performed using SPSS 21.0 (SPSS Inc., Chicago, IL, USA). Basic descriptive statistics were measured including the means, standard deviations, ranges, and percentages. The normality of the distribution was examined by the Kolmogorov–Smirnov test. Mean values between two independent groups were compared by the Mann–Whitney U test for continuous variables and by the χ*^2^* test for categorical parameters; comparisons between more than two subgroups were performed by ANOVA and Kruskal–Wallis h tests. Bivariate correlations were explored by Pearson’s (continuous variables) or Spearman’s (categorical variables) coefficients. Logistic regression analysis was performed as multivariate analysis on parameters with significant differences observed in the univariate analysis. The ability of UA to predict NAFLD was evaluated using the receiver operating characteristic (ROC) curve analysis. The recommended cut-off value of UA for the optimum sensitivity and specificity ratio of the diagnostic test was determined. Differences were considered statistically significant if the two-tailed 𝑃 value was less than 0.05.

## 3. Results

There were 367 patients in total (225 patients in NAFLD group and 142 in control group). The mean BMI of the patients was 27.25 ± 4.02 in the NAFLD group and 24.71 ± 3.34 in the control group. The mean age of the patients was 34.08 ± 9.08 years in the NAFLD group and 34.24 ± 8.72 years in the control group. There were 187 males (61.4%) in the NAFLD group and 81 males (57.1%) in the control group. Table 1 presents a comparison of the clinical, laboratory, and demographic data of the NAFLD and control groups. The total bilirubin, albumin, GGT, and BUN values were similar for both groups. The BMI, AST, ALT, ALP, TG, TC, HOMA-IR, UA, and creatinine values of the NAFLD group were found to be significantly higher than those of controls. A positive correlation was found between the NAFLD stage and UA and creatinine values. On the other hand, BUN values did not show any correlation with the NAFLD stage (Table 2).

To evaluate the independence of associations between NAFLD and anthropometric and biochemical parameters, we undertook a regression analysis with NAFLD/non-NAFLD as the binary outcome (Table 3). This analysis showed that the following factors were independently associated with NAFLD: BMI, HOMA-IR, GGT, and UA.

**Figure 1:**
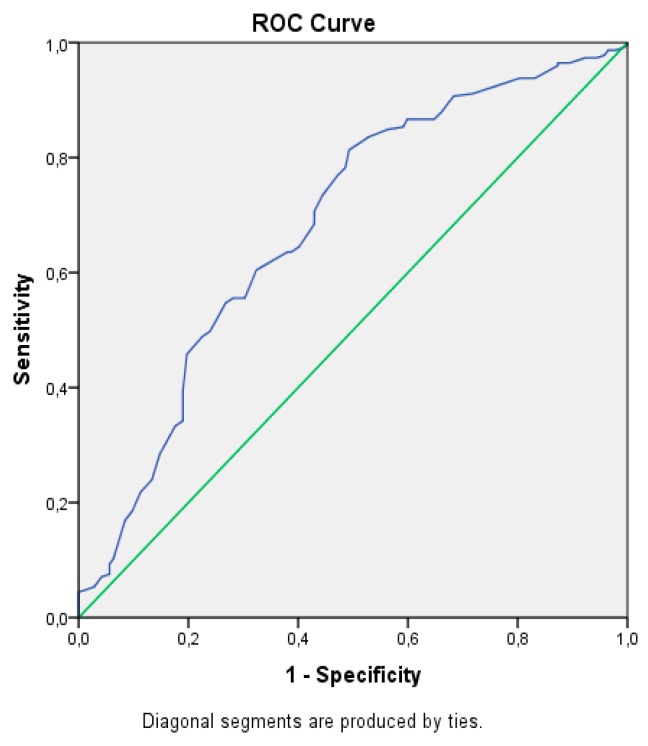
Curve for the prediction of NAFLD by uric acid.

In addition, a comparison of the mentioned variables between three groups (control, NAFLD group I, and NAFLD group II) is presented in Table 4. The Kruskal-Wallis test showed that UA and creatinine values were significantly different among the three groups. The BUN value was similar for the three groups. The UA value was significantly different between NAFLD group I and the control group, between NAFLD group II and the control group, and between NAFLD groups I and II. The creatinine value was significantly different between NAFLD group I and the control group and between NAFLD group II and the control group, but it was not different between NAFLD group I and NAFLD group II. No statistically significant difference was detected regarding the BUN value between NAFLD group I and the control group, between NAFLD group II and the control group, and between NAFLD groups I and II (Table 4). The ROC curve for UA in estimating NAFLD was constructed, and an area under the curve of 0.682 was found (Figure 1). The cut-off value of UA was 4.75 mg/dL with a sensitivity of 45.8% and a specificity of 80.3%.

## 4. Discussion

With this study, we found that in non-obese and young patients with NAFLD, UA levels were significantly higher than those of individuals in a healthy control group. The UA level also showed a positive correlation with the degree of hepatic steatosis and NAFLD. Furthermore, UA was found to be an independent risk factor for NAFLD. In addition, creatinine levels were significantly higher than those of individuals in the healthy control group, but the difference in the BUN level between groups was insignificant.

Nutritional, metabolic, and genetic factors contribute to the development of NAFLD. NAFLD is among the most common causes of chronic liver disease worldwide. It was shown to be the most important cause of chronic liver disease and cirrhosis in a recent large study [19]. Not only is NAFLD an independent risk factor for cirrhosis and hepatocellular carcinoma, but it can even be a risk factor for extrahepatic diseases such as type 2 diabetes and cardiovascular disease [20]. Oxidative stress, systemic inflammation, and insulin resistance are known risk factors for liver disease progression and NAFLD development [21]. HOMA, BMI, TG, TC, and UA are known to be associated with NAFLD [22,23,24,25]. Likewise, in our study, we also found that HOMA, BMI and TG, TC, and UA levels were different between NAFLD patients and individuals in the control group.

UA is an end product of purine metabolism. Its level in blood can increase with insulin resistance, atherosclerosis, hypertension, and obesity. The factors resulting in this relationship are hypothesized to be inflammation and oxidative stress [9,26]. Moreover, an increasing amount of data suggests a link between UA and NAFLD. Many recent observations were done in order to elaborate on this connection [8,9,13,26,27,28,29]. It can be said that this connection is strengthened by studies that used xanthine oxidase inhibitors on animals in order to decrease UA production. These studies concluded that this usage of xanthine oxidase inhibitors resulted in reduced progression of NAFLD [15,30,31]. Many hypotheses were put forward. However, the pathogenesis still remains unclear. One hypothesis is that there is a link to UA-induced oxidative stress in adipocytes and vascular cells and inflammation via the production of p38 mitogen-activated protein kinases and cyclooxygenase-2 by UA. UA might also increase in the presence of expression, leading to the amplification of the lipogenic effects of fructose, causing triglyceride accumulation in hepatocytes [30,32].

In our study, we found that UA levels were higher in NAFLD patients than in the control group, and we also found a positive correlation between the NAFLD stage and UA. Similar findings were reported in some studies [22,24]. However, there are some limitations in these studies, including the selection of patients (diabetic and obese patients) and the method of diagnosis of NAFLD (diagnosed with ultrasonography). However, in our study, NAFLD was diagnosed by biopsy, and our study included non-obese non-diabetic patients.

Many studies showed that the elevation of UA levels is an independent predictor of an increased risk for NAFLD [24,25,33]. Similarly, in our study we found that the elevation of UA levels is an independent predictor of increased risk for NAFLD. We estimated a cut-off value for UA for the prediction of NAFLD of 4.75 mg/dl, while two other studies found a cut-off value for UA of >6 mg/dl [22,24]. This might be due to the selection of the patients. Both of these studies included diabetic and obese patients and had small sample sizes [22,24]. Therefore, the results of our study might be more powerful.

BUN and creatinine are parameters that are used to predict kidney function in daily practice [34]. Similar to our research, many researchers have concluded that there is no correlation between BUN and NAFLD [30,33]. In contrast to our study, some authors argued that patients with NAFLD have higher BUN values [35,36]. In our study, we also found a correlation between the creatinine level and NAFLD. Thus, whether a relationship exists still remains unclear. Hence, more research on larger cohorts must be made.

To date, the association between UA and NAFLD has been demonstrated in many clinical studies. However, in these studies, UA levels were over the normal range [22,24]. Different from these studies, the UA levels of our patients were in the normal range but were significantly higher than those of individuals in the control group. From a theoretical perspective, according to our findings, we hypothesize that UA stimulates fat accumulation, hepatic steatosis, and hepatic inflammation, factors that are crucial in the pathogenesis of NAFLD.

### Limitations

A possible limitation of our study may be a reporting or recall bias caused by patient histories, most of which reported a relatively healthy diet. The retrospective aspect of the study and the retrieval of data only from records may be limitations of the study. Finally, in our study, the BMI values of patients with NAFLD were significantly higher than those in the control group. However, differing from published studies, we excluded obese patients with BMI values of 30 and over. Therefore, the BMI difference between the control and NAFLD groups might not have affected our results.

## 5. Conclusions

In summary, the UA level was found to be significantly and independently associated with hepatocellular steatosis and the NAFLD stage in biopsy-proven NAFLD patients. UA is a simple, non-invasive, cheap, and useful marker that could be used to predict steatosis in patients with NAFLD. In addition, we suggest that caution should be exercised in terms of NAFLD progression when the UA level is greater than 4.75 mg/dl. Further investigations in larger prospective studies are needed to validate our study results.

## Figures and Tables

**Table 1:** Clinical, laboratory, and demographic data of non-alcoholic fatty liver disease (NAFLD) patients compared with controls.

Characteristics of Patients	NAFLD (n = 225)	Control (n = 142)	P*
Age	34.24 ± 8.72	34.08 ± 9.08	0.80
Gender (female/male. n)	87/138	61/81	0.41**
Body mass index (kg/m2)	27.25 ± 4.02	24.71 ± 3.34	<0.001
ALT (IU/L)	22.76 ± 14.13	18.47 ± 10.88	0.001
AST (IU/L)	18.48 ± 5.66	16.99 ± 4.60	0.015
ALP (U/L)	72.92 ± 24.40	66.11 ± 20.06	0.009
GGT (U/L)	20.72 ± 15.76	18.23 ± 12.33	0.10
Total Biluribin (mg/dl)	0.59 ± 0.31	0.59 ± 0.53	0.17
Albumin (g/dll)	4.68 ± 0.31	4.66 ± 0.61	0.10
Triglyceride (mg/dL)	117.74 ± 66.10	96.54 ± 43.79	0.03
Total cholesterol (mg/dl)	185.79 ± 41.36	178.90 ± 45.13	0.03
HOMA-IR	2.60 ± 1.61	1.71 ± 0.77	<0.001
BUN (mg/dl)	12.18 ± 3.64	12.04 ± 3.13	0.32
Creatinin (mg/dl)	0.79 ± 0.16	0.69 ± 0.16	<0.001
Uric acid (mg/dl)	4.36 ± 1.36	3.48 ± 1.30	<0.001

ALT: alanine aminotransferase; AST: aspartate aminotransferase; ALP: alkaline phosphatase; GGT: gamma-glutamyl transferase; HOMA-IR: homeostatic model assessment insulin resistance; BUN: Blood Urea Nitrogen. * Mann–Whitney Test, ** *χ*^2^ Test.

**Table 2:** Correlation analysis between biochemistry parameters (uric acid, creatinine, blood urea nitrogen) and NAFLD stage.

Biochemistry Parameters	NAFLD Stage	
	r	P*
Uric Acid	0.338	<0.001
Creatinine	0.243	<0.001
Blood Urea Nitrogen	0.069	0.185

* Spearman Correlation.

**Table 3:** Independent association of variables with NAFLD (logistic regression analysis) *.

Independent Factors	Odds Ratio	95% Cl	*P* Value
Age	0.987	0.956–1.018	0.405
Gender (female/male, %)	1.602	0.848–3.025	0.147
Body mass index (kg/m2)	1.084	1.001–1.174	0.047**
ALT (IU/L)	1.020	0.984–1.057	0.289
AST (IU/L)	1.016	0.943–1.094	0.678
ALP (U/L)	1.010	0.998–1.022	0.114
GGT (U/L)	0.972	0.949–0.994	0.015**
Total Biluribin (mg/dL)	1.002	0.556–1.809	0.994
Albumin (g/dL)	0.935	0.511–1.712	0.829
Triglyceride (mg/dL)	1.002	0.997–1.007	0.479
Total cholesterol (mg/dl)	1.001	0.994–1.007	0.808
HOMA-IR	1.743	1.248–2.434	0.001**
BUN (mg/dl)	0.962	0.888–1.043	0.351
Creatinin (mg/dl)	38.636	5.738–260.146	0.000**
Uric asit (mg/dl)	1.592	1.299–1.951	0.000**

ALT: alanine aminotransferase; AST: aspartate aminotransferase; ALP: alkaline phosphatase; GGT: gamma-glutamyl transferase; HOMA-IR: homeostatic model assessment insulin resistance; BUN: blood urea nitrogen. * Models of logistic regression between NAFLD as the outcome and uric acid (UA) concentration, and age, gender, anthropometric, and biochemical parameters as variables. NAFLD (= 1) and non-NAFLD (= 0) were evaluated. ** P < 0.05.

**Table 4:** Comparison of biochemistry parameters between NAFLD groups I–II and the control group.

Biochemistry Parameters	Group I (n = 201)	Group II (n = 24)	Control (n = 142)
Uric Acid	4.28 ± 1.32*	5.07 ± 0.78**	3.48 ± 1.36
Creatinine	0.79 ± 0.16*	0.78 ± 0.16*	0.69 ± 0.16
Blood Urea Nitrogen	12.13 ± 3.21	12.58 ± 2.43	12.04 ± 3.64

* P < 0.05 versus Control; ** P < 0.05 versus Group I and Control.
